# Spicy Personality: On the Relationship Between Personality Traits and the Preference for Spicy Foods

**DOI:** 10.3390/foods15030559

**Published:** 2026-02-04

**Authors:** Ceyhun Uçuk, Charles Spence

**Affiliations:** 1 Gastronomy and Culinary Arts Department, Gaziantep University, University Boulevard, Gaziantep 27100, Türkiye; ceyhunucuk@gmail.com; 2 Department of Experimental Psychology, University of Oxford, Life and Mind Building, South Parks Road, Oxford OX1 3PS, UK

**Keywords:** pungency, personality, taste preferences, spices

## Abstract

This narrative historical review assesses the relationship between personality traits and the preference for spicy foods. While genetic, cultural, and personality factors have all been shown to influence taste preferences, the evidence that has been published to date suggests that personality plays a greater role in the liking and consumption of spicy food than for those basic tastes linked to the essential elements of a healthy diet. Archaeological and historical data illustrate the global dissemination and cultural integration of *Capsicum* into the human diet. Meanwhile, physiological and psychophysical research highlight that the pungent quality of capsaicin, together with the gustatory and olfactory cues associated with the flavour of chilli, affects hedonic evaluation, with repeated exposure often increasing acceptance through a process of desensitisation. Developmental factors, such as prenatal taste/flavour transmission and benign risk learning during childhood, underpin adult preferences. Cross-cultural studies reveal that the tolerance for pungency varies by country/culture and is also markedly shaped by personality traits. Recent social media trends have also increased some people’s exposure to very spicy foods, linked to their sensation-seeking tendencies. As such, those theories that focus solely on biological sensitivity and cultural exposure likely fail to capture personality-driven factors like sensation seeking and reward sensitivity that drive the liking for spicy foods.

## 1. Introduction

Humans are the only species that deliberately opt to ingest substances that may cause them pain, such as capsaicin-containing chile/chilli peppers. According to Cordell and Aroujo [1], one in four individuals globally consume hot peppers on a daily basis, and that figure is likely to be even higher today. As such, it becomes increasingly important to understand the psychological and cultural motivations behind this popular habit. Why, one might ask, do so many of us voluntarily welcome painful stimuli into our daily diets? According to the research, this unusual inclination has a variety of causes, and may be influenced by everything from culture and personality, through to genetics and prior exposure. This narrative historical review (see [2,3], on the strengths of narrative reviews) examines the correlation between personality traits and the predilection for spicy foods. Multiple studies now indicate that the variation in people’s preference for spicy foods is shaped by multiple factors, including genetic predispositions, psychological characteristics, as well as behaviours that are culturally determined [4,5,6,7,8,9,10,11,12]. Comprehending the various factors that influence the consumption of spicy foods is essential for those wishing to examine the consumer’s interaction with spicy food in both private and social contexts (e.g., as in the various extreme online eating challenges that have sprung up in recent years; see [13,14,15,16]).

### 1.1. Genetic Underpinnings of the Preference for Spicy Food

Genetic variables significantly influence taste perception, especially with regard to spicy foods with capsaicin, the compound responsible for the experience of heat in chile/chilli [8,10,11,17,18,19,20,21]. Genetic differences affect the perception of spiciness and thereby shape people’s preferences for spicy foods [4,5,7]. Moreover, those individuals who possess genetic polymorphisms, such as to TRPV1 (Transient Receptor Potential Vanilloid 1/capsaicin receptor), may exhibit an increased sensitivity to capsaicin. This, in turn, could result in either a heightened intake or an aversion to spicy foods. These divergent pathways appear to depend on both biological sensitivity and learning/prior experience, which affect both emotional and motivational responses to capsaicin [5,6,7,12].

**Figure 1:**
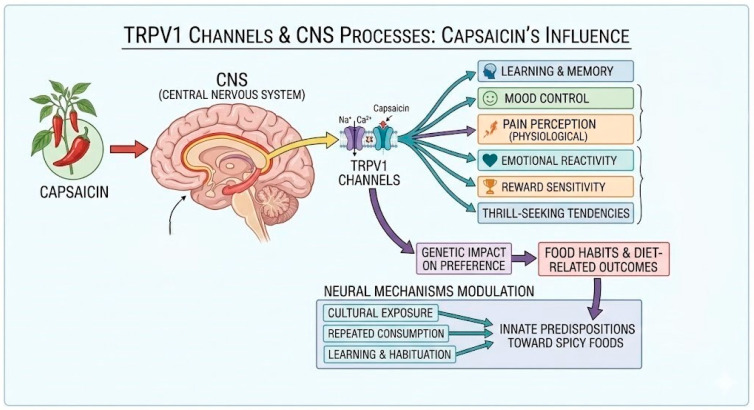
Schematic representation of capsaicin-induced TRPV1 activation and associated central nervous system processes [5,6,12,21,22].

TRPV1 channels may contribute to central nervous system processes, including learning, memory, and the control of mood. The channels activated by capsaicin can affect the physiological perception of pain as well as personality-linked psychological responses, including emotional reactivity, reward sensitivity, and people’s thrill-seeking tendencies [22]. The genetic impact on preference may extend well beyond taste perception, influencing food habits and diet-related sensory and physiological outcomes across the individual’s lifespan. Cultural exposure and repeated consumption experiences have been shown to modulate the neural mechanisms that underlie capsaicin tolerance, suggesting that learning and habituation can reshape the consumer’s innate predispositions toward spicy foods (see Figure 1) [5,6,12,23].

The physiological increase in arousal that results from the experienced heat associated with the consumption of chile (chemesthesis; specifically the TRPV1-mediated burn of capsaicin) influences various behaviours related to spicy food (approach-avoidance, desire, consumption frequency) more robustly than do reactions to the so-called ‘basic’, or primary, tastes (sweet, sour, salty, bitter, and umami [24,25,26,27]). This chemesthetic response is mediated by the trigeminal system, which, in turn, indirectly influences certain taste responses through its interaction with gustatory perception [28,29,30]. Variations in sensitivity to spicy heat as well as personality characteristics have been shown to affect patterns of food intake [31,32,33,34,35,36]. Moreover, repeated exposure and cultural learning also modify people’s hedonic assessment of spicy foods [37,38,39,40,41,42,43]. Such findings therefore indicate that preferences for spicy foods are influenced by multiple sensory and psychological factors well beyond issues related to the basic perception of taste. Moreover, the integration of flavour in the brain (taste, smell, and, when present, chemesthesis) is influenced by learning, experience, and attention. Therefore, any trigeminal inputs tend to exert a more significant impact on our perceptions and actions than would be expected from a consideration of the gustatory input by itself [44]. Prescott [45] further illustrated the significant role of the trigeminal system in food perception, asserting that this sense plays a crucial role in shaping our flavour experiences.

In the present review, capsaicin is discussed chiefly within the framework of trigeminal chemesthesis. Its chemogenic characteristics-namely, burning, stinging, and heating-are induced through TRPV1 activation in the oral and nasal cavities. These sensations influence sensory-emotional responses and function as a crucial element of multisensory flavour perception. It is important to note, though, that capsaicin-driven trigeminal inputs also interact with the gustatory and olfactory systems. For example, the consumption of capsaicin has been shown to alter taste thresholds, shape aroma perception through saliva-related and trigeminal-olfactory interactions, and influence textural perception, including the discrimination and oral thickness [25,43,46,47]. The sensations of ‘burning’ and ‘stinging’ experienced while consuming spicy foods arise from the activation of trigeminal nerve endings in the oral cavity and nasal passages by chemical substances. During this process of trigeminal activation, capsaicin stimulates TRPV1 channels, producing a burning sensation [48]. Trigeminal chemical sensations are not solely painful; they also constitute a component of a multisensory experience that accompanies, and may be considered as part of, the perception of taste and flavour. Clinical and non-clinical data demonstrate that intranasal trigeminal sensitivity is quantifiable, with capsaicin-based (TRPV1-agonist) intranasal stimuli being characterised by sensations such as burning, tingling, and stinging [25,49,50].

Individual variations in trigeminal activation in response to stimulation by capsaicin are attributed to both biological sensitivity, including the perceived intensity of burning and stinging sensations (chemesthetic response) and TRPV1-dependent desensitisation, as well as by learning and the dynamics of habituation. Furthermore, personality traits account for a substantial portion of the variance in both people’s preference for, as well as their frequency of consumption of, spicy foods. In particular, sensation seeking and reward sensitivity have been shown to correlate with a greater liking for spicy foods and more frequent chilli intake. Additionally, gender-related variation is evident, with men tending to report higher sensation seeking and a greater acceptance of spicy foods than women [13,51].

Personality-based differences in the decision to consume spicy food likely demonstrate, at least in part, fundamental differences in sensory processing and trigeminal activation [51,52]. This sensory-biological variability helps to explain how trigeminal stimulation can help to either enhance or diminish multisensory flavour perception, contingent upon the food matrix and stimulus intensity, thus helping to explain why it is that pungency elicits hedonic appeal in some individuals while inducing aversion in others [37,43]. Such personality-dependent divergence aligns with evidence suggesting that trigeminal stimulation can shape cognitive-emotional responses to pungency which, in turn, relate to behavioural tendencies such as risk-seeking [53,54].

Trigeminal activation plays a foundational role in the multisensory perception of taste, as flavour is shaped jointly by gustatory, olfactory, and chemesthetic inputs (should the latter happen to be present). Chemesthetic sensations such as warmth, burning, and tingling contribute distinct sub-qualities that help to define the perceptual profile of those foods that are described as pungent. Moreover, individual variations in chemesthetic sensitivity have been shown to modulate the perception of pungency through crossmodal interactions (e.g., TRPV1/TRPA1 pathways influencing flavour signaling), helping to explain why it is that some individuals experience a desire for pungent or spicy foods whereas others exhibit avoidance instead [11,26,39,55].

Variations in chemesthetic sensitivity among individuals extend beyond biological factors; they also directly affect people’s pleasure and consumption as far as spicy and pungent foods are concerned. The habitual intake of pungent substances may diminish the sensation of burning while, at the same time, enhancing their hedonic acceptance [56]. The trigeminal burning sensation has been shown to inhibit taste and olfactory perception in certain situations, while amplifying the experience of flavour in others [57]. The chemical burn induced by capsaicin and related pungent compounds is regarded as a fundamental component of flavour (at least when it is present), alongside taste and aroma, significantly influencing both sensory pleasure and culinary preferences [28,58,59].

### 1.2. Personality and Cultural Influences on the Preference for Spicy Food

Cultural norms and social learning further shape the individual sensitivity to spice tolerance and chemesthetic stimuli. A comparative analysis reported by Trachootham et al. [60] revealed that those consumers from Thailand exhibited a greater desire for spicy and chemesthetic sensations and consume pungent foods more frequently, whereas Japanese participants exhibited a lower preference and consumption frequency for spicy-pungent stimuli. This pattern of cultural variation is also reflected in Thai cuisine, which is distinguished by the extensive use of chile and other spicy ingredients and has gained significant global recognition for its characteristic spiciness [61,62].

In Mexico, according to Rozin and Schiller [62], chilli eating is predominantly a cultural legacy associated with machismo attitudes. At the same time, however, it is also important to recognize that it is deeply embedded in Mexico’s traditional food culture, which spans everyday home cooking, regional culinary practices, and long-standing agricultural-culinary traditions [11,63,64,65,66]. Conversely, in the United States, the inclination towards chilli has been linked to sensation-seeking and having a bold attitude, indicating more of a personality-driven, rather than a cultural, rationale. Byrnes and Hayes [51] indicated that elevated ratings of sensation-seeking amongst those from the United States were positively associated with the consumption and desire for spicy peppers. Meanwhile, Wang et al. reported that an affinity for spicy tastes/flavours in China correlates with heightened risk-taking behaviours and also showed through laboratory research that consuming spicy food temporarily enhances the inclination to engage in risky activities. Such behaviours include selecting disadvantageous high-reward/high-penalty decks (A/B) in the Iowa Gambling Task (IGT) and endorsing gambling-and recreation-related risks on the Domain-Specific Risk-Taking Scale (DOSPERT). Wang et al. propose that this establishes a congruent psychological profile characterised by spice consumption, adventurousness, and a receptiveness to novel experiences [47,53].

**Figure 2:**
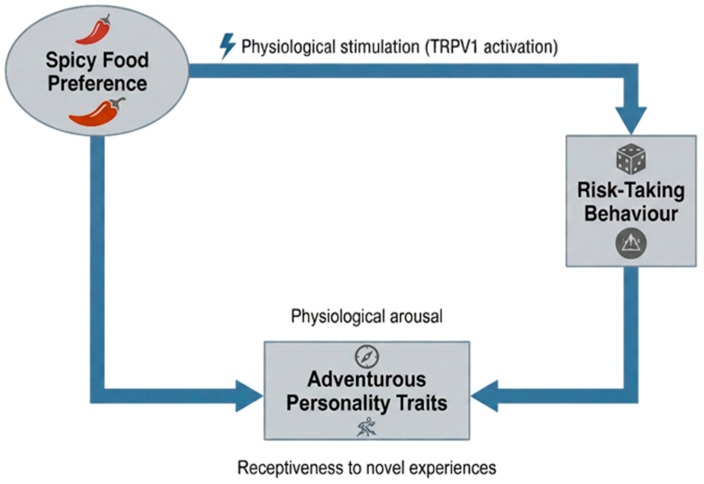
Conceptual framework of the relationship between a preference for spicy food, adventurous personality traits, and risk-taking behaviour.

Byrnes and Hayes [67] highlighted the existence of a correlation between the preference for spicy food and risk-taking inclinations; however, behavioural assessments such as the modified Balloon Analogue Risk Task (mBART) failed to reveal a direct connection (see Figure 2). Meanwhile, Fu et al. [14] proposed that the ingestion of spicy peppers may correlate with enjoyment as well as with risk-taking, sensation-seeking, aggressive ideas, and emotional states. Khajarern [68] discovered that the emotional states that were elicited by various movie genres led to reduced emotional reactions when consuming spicy (Tom Yum) and barbecue-flavored popcorn, than when consuming other flavours. Nonetheless, these flavours produced markedly elevated thirst ratings because of their spicy characteristics.

In a cross-cultural study conducted in the United States, Korea, and Denmark, Kim et al. [69] found that consumers’ acceptance of hot sauces was affected by their cultural background and motivated by hedonic factors. In particular, these researchers showed that Koreans and Danes preferred sweeter and more balanced sauces (GS, KS), whereas U.S. consumers showed a more polarized pattern and often favoured hotter sauces such as Sriracha, indicating that acceptance differs according to culturally shaped flavour expectations and perceived sauce-food appropriateness. Zhang et al. [70] found that Sichuan pepper enhances the impression of saltiness, but this impact was contingent upon individual sensitivities to chemesthetic stimulation and olfactory intensity as well as personality characteristics (e.g., sensation seeking, openness to experience, or tolerance for sensory novelty). Similarly emphasizing the interplay of genetic and psychological traits and cultural practices, Siebert et al. revealed that hot peppers exhibit greater acceptance rates in Mexico than in the United States. Recently, Chon and Kim [71] categorized consumers into spicy-tolerant and spicy-sensitive groups, thus demonstrating that tolerance correlates with a heightened preference for spicier kimchi, while at the same time acknowledging that cultural dietary practices may exacerbate such differences.

Andaleeb et al. [72] demonstrate via a cross-cultural sensory evaluation that cultural background and prior exposure to spice mixes shape both people’s hedonic reactions and their perceptual profiles. These perceptual profiles, which refer to the specific flavour and aroma attributes participants selected during evaluation using a Check-All-That-Apply (CATA) procedure (e.g., clove, cinnamon, Japanese curry flavour), differed systematically across cultural groups. In particular, Chinese customers evaluated the Chinese 5-spice samples more favourably, associating them with qualities such as ‘aromatic’ and ‘sweet’, while Pakistani consumers had a greater preference for samples of garam masala, correlating them with descriptors such as ‘spicy’ and ’strong’. The two groups exhibited divergent emotional responses, with Pakistani individuals indicating a greater positive affect towards familiar spice combinations. This study demonstrates that cultural environment systematically influences consumer perceptions and interactions with hot and spiced foods. Tolerance and preferences for spicy flavours varied markedly among individuals from diverse geographical and cultural backgrounds [73]. What the research reviewed in this section therefore demonstrates is that cultural experience functions as a primary lens through which consumers perceive, categorise, and appraise spiced foods including chile.

### 1.3. Sensory and Behavioural Mechanisms Shaping the Perception of Spicy Foods

Parker [74] was one of the first researchers to elucidate the trigeminal system’s response to specific chemical stimuli, resulting in sensations of burning and pain. Han, Müller, and Hummel [24] proposed that even though trigeminal stimulation is processed through neural pathways that are distinct from the taste receptors, it can nevertheless affect gustatory perception by modifying taste-transduction mechanisms at both the peripheral and central levels. Their study demonstrated that peri-threshold capsaicin decreased taste recognition thresholds, suggesting that trigeminal inputs may improve taste sensitivity without directly activating taste receptors. Yu et al. [75] demonstrated that trigeminal neurones respond to chemical stimuli; specifically, capsaicin activation elicits burning and pain sensations, thus confirming that spiciness represents a distinct sensory modality (chemesthesis), albeit one that is closely related to taste [24,27].

Rozin and Schiller [62] and Stevenson and Prescott [76] present empirical evidence for the behavioural mechanisms that explain desensitisation and sensation-seeking in the eating of spicy foods. Rozin and Schiller demonstrated that repeated exposure and culturally reinforced learning may transform the initially aversive sensation of chilli burn into a positively valued experience, demonstrating a clear hedonic reversal. Conversely, Stevenson and Prescott reported that prior sensory experience affects perceived burn intensity, with routine consumers rating identical capsaicin stimuli as less intense due to long-term experiential calibration. Collectively, these data elucidate how repeated exposure, learning, and experience influence the perception and consumption of spicy foods. Similarly, Endrizzi et al. [77] noted that individual differences in general pain sensitivity contribute to variations in capsaicin-induced (chilli) burn, and that these responses may be modified by repeated exposure.

Recent experimental research indicates that the liking for spicy foods may embody not just personality characteristics but also contextual emotional processes. Changes in stress-induced sensory motivation have been demonstrated to selectively enhance the appetite for intensely stimulating oral sensations such as spiciness, while diminishing the preference for sweetness and saltiness. The findings indicate that capsaicin-rich meals may serve as mood-regulating stimuli during heightened emotional arousal, offering temporary respite or stimulation through trigeminal activation. These situational mechanisms provide a crucial contextual framework for comprehending why persons with heightened emotion-seeking or reward sensitivity, especially in emotionally intense or stressful circumstances, are more attracted to hot meals [78].

In addition to examining the biological and sensory bases of spiciness perception, it is crucial to address the psychological factors that affect individuals’ reactions to pungent stimuli. At the same time, however, it is also important to recognize that the research that has been published to date often depends on limited samples, such as university students, young adults, or those individuals from a singular cultural background, and uses narrow methodological approaches (e.g., self-report surveys), thus restricting the generalizability of the results and so perhaps impeding a more thorough understanding of how these relationships differ across cultural, biological, and social contexts [79,80].

The common WEIRD (Western, educated, industrialised, rich, democratic) sampling bias in much of the psychological and behavioural sciences research highlights the necessity for cross-cultural analysis of perceptual and emotional processes [81,82]. Recent studies indicate that fundamental human cognitive attributes, including perception, attention, memory, and motivation, can all be influenced by culture [81,82]. The body of research reviewed here consolidates the growing empirical evidence connecting individual differences in spicy food preference and consumption with personality traits, while suggesting avenues for more inclusive and culturally informed future work. Expanding on these theoretical underpinnings, the subsequent sections integrate historical and modern viewpoints on personality-spiciness connections.

## 2. Theoretical Background

### 2.1. The History of Chili Pepper Consumption

Historically, trade and cultural interactions have impacted exposure to spices, thus influencing hedonic and evaluative responses to their taste/flavour. Additionally, empirical evidence indicates that these responses can be influenced by individual personality traits—specifically sensation seeking and reward sensitivity—which positively correlate with the preference for, and consumption of, spicy food [51,54,83]. Recent research has shown that cultural familiarity and consumption habits modulate the perceptual and hedonic assessment of spiciness, resulting in systematic differences between tolerant and sensitive consumer groups [71]. The domestication and global spread of chili peppers stand as a notable case of plant–human coevolution. The evidence indicates that the domestication of *C. annuum* commenced in Mexico, specifically in the northeastern and central-eastern areas, aligning with the Tehuacán and Ocampo discoveries [84]. Archaeological and paleoethnobotanical evidence indicates that early *Capsicum* use occurred in coastal Andean areas beyond Mesoamerica, implying that the geographical distribution of the genus’ most recent common ancestor encompassed the northern Andes—specifically Venezuela, Colombia, Ecuador, and Peru—with natural ranges extending along the Caribbean-Central America axis (see [85,86,87]. The Columbian Exchange later facilitated the introduction of chile peppers (and thus exposure to the burn of capsaicin) to the Old World, where it rapidly became integral to diverse cuisines [88].

Studies indicate that cultivated forms of *Capsicum* species derive from wild progenitors through human selection [89], while transcriptomic and genomic analyses reveal regulatory changes in fruit size, ripening, and capsaicinoid biosynthesis that distinguish domesticated peppers from their wild relatives [90]. The example of chilli peppers therefore provides a particularly intriguing example of how a locally domesticated plant can develop into a crop of worldwide cultural and commercial significance.

With the onset of the Modern Era, hot peppers (*Capsicum* spp.), native to the Americas, became disseminated swiftly across Europe, Asia, and Africa beginning in the 16th Century. In Mexico, they have assimilated into a long-established pattern of consumption that has persisted for millennia, whereas spices such as black pepper and ginger have become essential components of the daily diet in Southeast Asia [91,92,93,94,95]. This historical dissemination has given rise to worldwide distinctive ‘foodscapes’, enabling geographically specific reactions to flavours and other chemesthetic stimuli. The extensive historical trade in black pepper and the long-standing culinary use of ginger across Asia highlight how certain spices can become embedded in food traditions, even when they do not become uniquely associated with any single culture [94,95,96].

**Figure 3:**
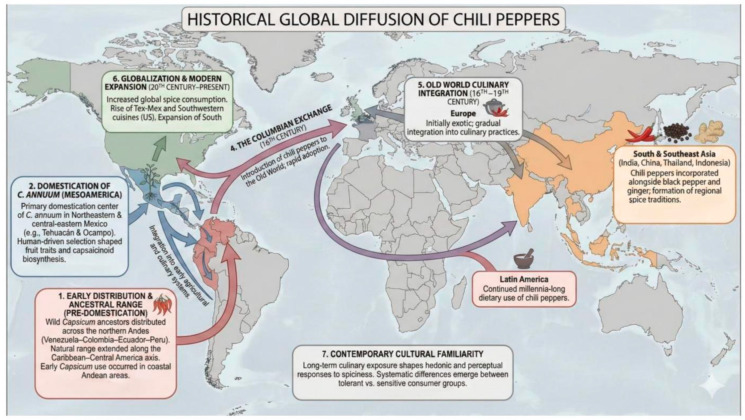
Global Historical Diffusion of Chili Peppers (*Capsicum* spp.) [10,11,71,84,85,87,88,89,91,94,100,101,102]. Arrows are colour-coded to indicate different types of diffusion processes. Blue arrows represent early regional spread and integration within the Americas prior to European contact. Red arrows denote the transatlantic transfer of chili peppers during the Columbian Exchange. Purple arrows illustrate subsequent intercontinental dissemination and integration into Old World culinary systems. Green arrows indicate modern global expansion and contemporary diffusion patterns.

Since the mid-20th Century, globalisation and commerce have augmented spice consumption in the West; the emergence of Tex-Mex and Southwestern cuisines in the US, together with the proliferation of South Asian cuisines in the UK, evidence this shift [11,30] (see Figure 3). It can be argued that the global historical development of spice consumption necessitates a reevaluation of mid-20th-century psychological studies concerning the link between personality and taste. Preliminary research conducted in the UK and North America in the 1960s and 1970s may unintentionally have overlooked non-Western experiences due to participants’ restricted familiarity at the time with hot peppers and strong spices [97]. Moreover, alongside cultural disparities, the historical experiences of societies with hot and spicy foods-such as the prevalence of peppers in the daily diet of Latin America compared to their long-standing status as an exotic commodity in Europe-have influenced contemporary individual taste responses. According to Van Bavel et al. [98], psychological factors need to be interpreted within a historical-cultural framework, aligning with the model put forward by Lewin [99], since behaviour emerges from the interplay between the individual and their environment.

### 2.2. Evolution of Chili Pepper Spiciness: Measurement, Mechanisms, and Cultural Adaptation

Scoville Hotness Units (SHU) serve as the principal metric for quantifying the spiciness of chilli peppers. The Scoville scale, established by American chemist Wilbur Scoville in 1912, relies on an organoleptic assessment whereby pepper extract was diluted with a sugar-water solution, measuring the number of dilutions necessary for tasters to no longer perceive any pungency [30,103,104]. This method, despite criticism for depending on the subjective assessment of pungency by human tasters, is acknowledged as the inaugural systematic and historically significant technique for detecting the presence of capsaicinoids (e.g., capsaicin and dihydrocapsaicin) [105,106,107,108,109,110].

Following on from Scoville’s innovative albeit subjective approach, the following decades were associated with significant developments in the measurement of pungency. Starting in the 1970s, measurement and standardization initiatives accelerated, leading to the use of analytical techniques including high-performance liquid chromatography (HPLC). The AOAC (Association of Official Analytical Chemists) method, facilitating the direct quantification of capsaicinoid concentrations and their conversion to Scoville Heat Units (SHU), was implemented by researchers [110,111], thus ensuring measurement uniformity. Simultaneously, the identification of the TRPV1 channel in 1997 clarified the mechanism by which capsaicin induces a burning sensation through the trigeminal system at a molecular level. TRPV1 has been identified as a polymodal ion channel responsive to various stimuli, including heat and acidity, and is linked to systemic functions such as pain perception and energy metabolism [22,112,113]. Clarifying this mechanism helps explain how repeated activation of the TRPV1 channel leads to sensory desensitization over time, while individual differences and expectation further modulate perceived spiciness [114].

The last fifty years of cultural dissemination have significantly altered patterns of hot pepper intake. Hedonic adaptation and increasing tolerance arise from repeated exposure to capsaicin-containing foods, with personality factors, especially sensation seeking, significantly influencing individual differences in perceived pungency, liking, and consumption patterns [51,115]. Frequent consumption of chilli peppers reduces capsaicin-induced oral burn over time due to desensitization, which can, in turn, increase tolerance and even enhance the pleasurable aspects of consuming spicy foods [27,100,101]. Historically, the consumption of spicy food in the West has been linked to notions of ‘masculinity’ and ‘machismo’ [63], but this association has evolved qualitatively with the proliferation of global cuisines. For instance, the emergence of ‘curry’ culture in Britain during the 1960s and the globalization of Mexican cuisine have transformed taste preferences [102,116,117,118].

Capsaicin is unique among pungent food compounds in having a dedicated psychophysical scale. Among commonly consumed trigeminal spices, *Capsicum* species exhibit the greatest natural variability in pungency, ranging from completely non-pungent *C. annuum* varieties to *C. chinense* cultivars exceeding 2 million SHU [119,120,121]. By contrast, other culinary irritants such as black pepper (piperine), ginger (gingerol), or Sichuan pepper (sanshool) show a far narrower range of potencies across botanical varieties [122,123]. Additionally, capsaicin elicits a distinctively intense TRPV1-mediated burning sensation that carries strong emotional, memory-related, and behavioural consequences [113]. These effects make it more salient perceptually than other chemesthetic stimuli and facilitate its incorporation into display-oriented or performative consumption practices [63,89,124]. When these properties of capsaicin are considered together, they clarify why chili peppers alone warranted formal psychophysical measurement, ultimately leading to the development of the Scoville scale [104,119].

**Figure 4:**
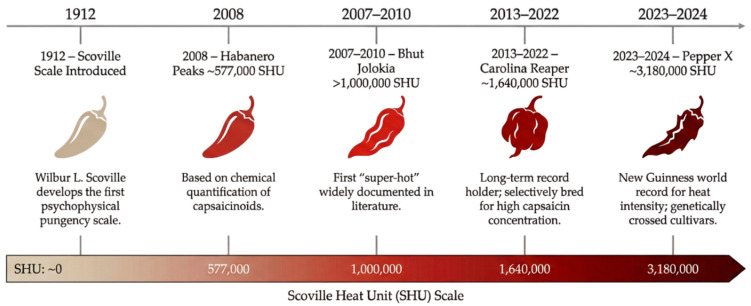
Evolution of extreme pungency in *Capsicum* cultivars according to the Scoville scale (1912–2024).

Since the Scoville scale was first introduced in 1912 [104], the pungency of cultivated *Capsicum* peppers has increased substantially through selective breeding. Early examples such as habanero-type *C. chinense* reached ~577,000 SHU [125], while by the early 2000s peppers like Bhut Jolokia had already exceeded 1,000,000 SHU [126,127], corroborated by studies on Naga Jolokia [128]. In the last decade, new elite cultivars have further expanded the scale’s upper limi-Carolina Reaper at ~1.64 million SHU and the current record-holder Pepper X approaching ~3.18 million SHU (see Figure 4) [129].

### 2.3. Social Media, Spectacle, and the Performative Turn in Spicy Food Consumption

The creation of a dedicated scale for capsaicin also helps position chili peppers as suitable candidates for contemporary spectacle-driven spicy-food culture, where high-capsaicin foods are frequently incorporated into competitive or performative contexts [124,130]. What once reflected personal or cultural eating practices has, in the contemporary era, become increasingly performative. That is, the appetite for sensation now unfolds within digital spaces, where social media amplifies these gustatory experiences (challenges) and transforms private sensations into public spectacle. Short-video and trend-oriented platforms enhance the visibility of ‘spicy challenges’ (e.g., the One Chip Challenge), thereby accelerating first-time participation and contributing to the normalization of high-capsaicin products [130,131,132].

Both experimental and observational research indicate that food-related information on social media can enhance preference, consumption, and brand attractiveness, particularly amongst children and adolescents [133,134,135]. For example, the frequency of watching mukbang/cookbang videos has been shown to correlate with an individual’s preference for high-arousal, energy-dense foods; the prominence of spicy and pungent ingredients in these subgenres [136,137] indicates that exposure may increase curiosity and pleasure-oriented motivation toward the tasting/consumption of pain-inducing spicy foods [138]. Perceived behavioural norms on social media—where users observe others engaging in ‘spicy challenges’—along with peer-endorsement can help to foster ‘pungency tolerance’ as a performance metric, interacting with individual sensation-seeking and reward sensitivity [51]. Collectively, social media increases exposure to spicy and pungent taste sensations and can stimulate curiosity- and pleasure- oriented motivations, both indirectly (through marketing and norm signaling) and directly (through participation in challenges).

## 3. Multisensory Determinants of Preference for Spicy Food

Extensive research conducted over several decades has demonstrated that individuals’ responses to spicy foods are influenced by a combination of physiological, psychological, and social factors [25,51,58,100,102,139,140]. In their preliminary investigation, Rozin and Schiller [62] made observations, conducted interviews, and experimental sensory assessments on two cohorts: one comprising 57 university students and Hispanic-Americans in the United States, and the other consisting of 63 participants from a hamlet in Mexico. Two hundred and sixty-five Mexican children also engaged in a brief preference assessment. In the experimental phase of the study, the participants were exposed to foods with escalating concentrations of capsaicin in order to assess their threshold, preference, and tolerance levels. Observations of familial dining in Mexico indicated that youngsters progressively adapt to the heat of chilli. The research incorporated insights from anthropology along with expertise provided by culinary specialists. The results indicated that Mexicans consume chilli three times a day, on average, whereas North Americans ate it only a few times weekly; yet, the majority in both demographic groups appreciated it. The findings indicate that although the consumption of chilli peppers may initially induce discomfort, frequent exposure can transform it into a learnt, or acquired, pleasure, which can be elucidated by the notion of “benign masochism”. Pramudya and Seo [141] asserted that cultural exposure to hot foods influences the development of tolerance, with consistent consumption diminishing perceived intensity and modifying hedonic assessment, rather than changing sensitivity itself.

Lawless and Stevens [142] explored interactions between chemical irritation and taste perception in controlled studies with 26 participants. Their participants initially gargled with liquids containing capsaicin (red pepper extract) or piperine (black pepper extract) before being administered solutions that exemplified the fundamental tastes of sweet, sour, salty, and bitter. The experiment was designed to examine the impact of chemical irritation on gustatory perception. The results highlighted a notable reduction in the perceived strength of sour (citric acid) and bitter (quinine) tastes, with no impact on the salty taste (The administration of piperine, however, led to more extensive reductions in the perception of all taste qualities). Lawless and Stevens concluded that capsaicin sensitivity shows substantial inter-individual variability, and that trigeminal irritation can suppress taste perception, although the magnitude of this inhibitory effect also differs across individuals.

Stevenson and Yeomans [143] categorized 32 adults into four groups according to their preference for chilli (likes/dislikes) and gender (female/male). Chilli enthusiasts, regardless of their gender, perceived low and moderate degrees of spiciness to be enjoyable, whereas chilli detractors had predominantly neutral reactions at these intensities. All of the groups assessed hotness at higher intensity levels unfavourably. Moreover, females experienced the same level of heat more intensely and assessed it more favourably than their male counterparts. The results indicate that originally neutral responses progressively transitioned to positive ones, resulting in the acquisition of a taste for hot peppers.

Törnwall et al. [144] investigated the genetic and environmental contributions of preference for spicy food among 331 twins (47 monozygotic, 93 dizygotic, and 51 identical twins). The participants in this study received a mild dose of capsaicin-infused jelly and were subsequently polled regarding their consumption of spicy foods and spices. Those individuals who reported disliking spicy foods experienced heightened perceptions of spiciness and deemed it to be less appetising, whereas those who enjoyed spicy foods provided more favourable assessments. The genetic distribution accounted for 18–58% of the variance, with the remainder of the discrepancies attributed to other causes. Moreover, the results suggest that sensitivity to capsaicin-induced spiciness has a heritable basis, and that shared genetic factors partly explain aversive responses, shaping stable long-term taste preferences.

Byrnes and Hayes [51] investigated the correlation between personality traits and preferences for spicy cuisine in a study with 97 participants aged 18–45 years (24 men) from a North American university and the local vicinity. The participants received strawberry jelly with differing levels of capsaicin and completed questionnaires regarding their sensory experiences. The results indicated a positive correlation between sensation seeking, sensitivity to reward, and the preference for, and intake of, spicy meals. Individuals with a substantial sensation seeking tendency demonstrated a preference for heightened pungency. The research indicates that individual variations in preferences for spicy food are influenced by both specific sensory tolerance for spicy and personality-driven tendencies.

Spinelli et al. [145] used the Sensitivity to Punishment (SP) and Sensitivity to Reward (SR) subscales of the SPSRQ to explore the relationship between personality traits and the perception and acceptance of spicy foods. Reward-sensitive populations typically pursue rewards and positive outcomes. High reward personalities may actively seek stimulating components in food (such as the burning sensation of chili peppers and the sour astringency of citrus beverages). Punishment-sensitive populations tend to avoid punishment and negative outcomes; they are more cautious, neurotically anxious, and inclined to avoid risks and uncertainty.

Nolden and Hayes [100] conducted a two-session experimental investigation including 82 adults (34 males, mean age 32 years) from a North American university and its local vicinity. The participants were categorised into low, medium, and high groups according to their liking for hot pepper dishes and intake frequency. Eight distinct doses of capsaicin solutions were assessed in the studies, with participants rating the severity of burning and spiciness, alongside their degree of hedonic preference. Frequent and high consumers rated the burning feeling from capsaicin as less severe and assessed it more favourably, whereas infrequent consumers encountered a more pronounced burning sensation. Moreover, liking scores were found to rise with an increase in the preferred level of spiciness. The research indicated that variations in capsaicin perception are associated with both sensory thresholds and sensory adaptation (desensitisation) processes related to dietary factors.

Kish [146] investigated the links between active and passive food choices, revealing that people’s dietary inclinations may be associated with biological elements, psychological characteristics, and behavioural tendencies. Kish and Donnenwerth [147] illustrated that the inclination to favour foods providing intense sensory experiences is associated not only with biological sensitivities or physiological requirements but also with personality traits and social influences, highlighting the impact of factors such as openness to new experiences, risk-taking propensity, and cultural background on food preferences.

Kono et al. [148] investigated the physiological and neurological impacts of capsaicin intake in 18 healthy individuals. Participants received capsaicin and various flavour solutions, and measurements were taken for salivation, secretory immunoglobulin A (SIgA), α-amylase, heart rate variability (HRV), and EEG activity. The results indicated that capsaicin enhanced salivation more effectively than flavour solutions, elevated SIgA and α-amylase levels, and resulted in a notable increase in beta-band EEG activity. The research indicated that capsaicin influences both sensory preference and the autonomic nervous system, as well as cognitive functions, underscoring the neurophysiological aspects of reactions to capsaicin. These physiological alterations suggest potential links between capsaicin responsiveness and individual differences in stress reactivity and emotional processing.

Prescott [149] examined the mechanisms of sensitisation (augmented burning sensation) and desensitisation (diminished sensation) related to capsaicin in two trials involving 14 adults. The initial experiment used capsaicin solutions of differing doses and impregnated filter sheets. Marked desensitisation was noted after a 10 min interval subsequent to short sensitisation at elevated doses. In the second experiment, the participants ingested soup and chilli con carne containing capsaicin. Despite the absence of severe sensitisation shown in the laboratory, desensitisation reemerged following the interval. The findings suggest that the perception of capsaicin is affected by physiological mechanisms and individual psychological traits.

Ludy and Mattes [115] went on to conclude that those individuals who enjoy spicy foods are more likely to have an extroverted personality type. Beyond behavioural adaptation, further research has investigated the neurological underpinnings of desensitisation to capsaicin, emphasising the manifestation of sensory habituation within the trigeminal system. Berry and Simons [150] reported that the burning sensation from capsaicin in oral areas diminishes with repeated exposure, signifying the onset of regional desensitisation. Neerven and Mouraux [151] demonstrated that repeated capsaicin stimulation of the trigeminal system is associated with diminished neuronal responses. Yang et al. [152] found that capsaicin exposure influences intensity perception and thresholds, hence corroborating the perceptual aspect of tolerance. Arora et al. [153] examined the pharmacological mechanisms of capsaicin, indicating that desensitization is crucial for analgesia and spicy food consumption behaviours. Taken together, these findings suggest that the hedonic appeal of spicy foods is influenced by a dynamic interplay amongst behavioural adaptation, neurophysiological changes, and personality-driven motivation.

## 4. The Evolution of Flavour (Or Taste) Preferences: From the Prenatal Stage Through to Childhood

Intermittent exposure to a food has been shown to facilitate the development of tolerance [154,155,156,157,158,159]. The principle of exposure-based learning extends postnatal experiences, with accumulating data indicating that taste and flavour preferences may commence prior to birth. Indeed, a mother’s dietary practices during gestation and lactation may exert a significant influence over her infant’s taste preferences. For instance, early research by Mennella, Jagnow and Beauchamp [160] established that certain volatile flavour compounds, specifically those ingested by mothers during gestation and lactation (e.g., carrot juice, garlic), are conveyed to the foetus via amniotic fluid and breast milk, resulting in infants exhibiting more favourable reactions to these aromas.

A review by Spahn et al. [161] highlights that maternal diet influences flavour transfer via amniotic fluid and breast milk, substantially affecting children’s taste preferences. Garner [162] indicates that prenatal and postnatal exposure to flavours significantly influences long-term infant preferences. Recent evidence also shows that maternal diet-derived odours reach the fetus and can program later metabolic and taste responses to food [163]. Wu and Suzuki [164] that parental high fat intake can exert intergenerational effects on offspring through potential epigenetic mechanisms. Ustun et al. [165] established that foetuses can display sensitivity to particular chemosensory stimuli (e.g., volatile flavour and fragrance chemicals) consumed by their mothers during gestation, and that even stimuli delivered in single-dose capsules might affect foetal behavioural responses. As such, the flavours and scents in a mother’s diet during pregnancy may affect children’s subsequent taste preferences. The uncertainty surrounding foetal taste abilities prompts the question of whether a preference for spicy foods might arise from early exposure to certain chemosensory signals, considering that hot pepper serves as a trigeminal oral and nasal stimulant. However, because most evidence on prenatal and early postnatal flavour learning concerns olfactory and gustatory cues transferred via amniotic fluid and breast milk [160,161], and capsaicin’s burn is mediated by a distinct trigeminal nociceptive system rather than taste or smell [59,166], the extent to which such early exposures modulate later chemesthetic responses to chilli peppers may be limited or at least remains uncertain.

Research from North America indicates that the consumption of hot peppers and spicy foods, which commences in modest quantities during childhood, tends to increase with age, notably influenced by sensory adaptation, favourable associations, and the perception of ‘harmless risk’. Logue and Smith [167] conducted a survey including over 303 people aged 14 to 68 years in the United States. Their results revealed a positive correlation between the desire for hot or high-risk foods and sensation seeking, while a negative correlation was shown for sweet or bland, low-risk foods.

Rozin, Ebert, and Schull [168] reported a study in which they presented crackers having differing levels of spiciness to their participants, documenting the latter’s hedonic assessments every 10 s for the initial minute and subsequently every 30 s, to assess temporal variations in hedonic responses. The findings revealed that the burn of hot peppers initially heightened taste awareness, but after roughly two minutes, only the solitary burning sensation remained, with its appeal varying according to an individual’s preference for chilli. According to Rozin [169], dietary preferences are a psychobiological phenomenon arising from both nutritional requirements and the interplay of learnt experiences, cultural traditions, and biological reactions. Rozin [169] expanding on this position, suggesting that the preference for the burning sensation induced by capsaicin is cultivated over time by repeated exposure, social reinforcement, and cultural conventions.

Subsequent physiological research has demonstrated that repeated exposure results in the development of tolerance to capsaicin. For example, a seminal study by O’Neil and colleagues [170] revealed that exposure to capsaicin induces a temporary enhancement in sensitivity, followed by considerable desensitization with repeated exposure. Green and Rentmeister-Bryant [171], meanwhile, established that capsaicin-induced desensitization is temporarily reversed (“stimulus-induced recovery”-SIR) when the subsequent stimulation matches or exceeds the intensity of the prior stimulus. Green and Hayes [24] discovered that capsaicin, piperine, and zingerone elicit taste sensations as well as trigeminal burning sensations via distinct pathways, indicating no direct association between gustatory intensity and burning sensation. The research demonstrates that repeated exposure leads to the development of tolerance to capsaicin. Stevenson and Prescott [76] similarly demonstrated that capsaicin alters flavour perception, as regular consumers exhibit modified sensory integration between taste and burn intensity. Breslin et al. [172] showed that repeated exposure to capsaicin may facilitate tolerance to its trigeminal burn through adaptive changes in trigeminal sensitivity.

Considering that repeated exposure alters both sensory and emotional reactions to capsaicin, this adaptation process necessitates an examination of its physiological foundations. Repeated exposure to capsaicin induces both peripheral and central desensitization, which modulates the hedonic experience of spice. Human psychophysical studies indicate that capsaicin-induced desensitization can shift taste perception, illustrating a bidirectional interaction between gustatory and trigeminal systems [173]. Neurophysiological recordings further show patterns of sensitization, desensitization and delayed recovery in brainstem trigeminal pathways [174], while frequent spicy food consumers display reduced sensitivity to capsaicin [169]. Together, these findings support the notion that tolerance and enjoyment of pungency are dynamically shaped by exposure-driven neural plasticity. Szolcsányi [175] characterised tolerance as a cyclical process including short-term sensitization followed by long-term desensitization within the trigeminal pathways, exemplifying a type of sensory adaptation akin to behavioural habituation. Ludy et al. [176] indicated that those who frequently ingest capsaicin experience a diminished burning sensation at identical doses, implying desensitization resulting from heightened frequency of exposure.

Physiological studies have elucidated that tolerance to capsaicin transcends mere perception, involving metabolic regulation, hunger, and personality-dependent enjoyment. Nolden et al. [26] established that repeated exposure to low-dose capsaicin over two weeks results in induced desensitization of mouth pain receptors in humans. The research demonstrated a diminished capsaicin response and cross-sensitization to alternative stimuli. The data indicate that capsaicin tolerance arises from both direct and indirect sensory adaptation mechanisms. Ludy and Mattes [115] observed that capsaicin intake can enhance thermogenesis, elevate energy expenditure, and diminish appetite, however these effects have been shown to fluctuate based on habituation and tolerance. Byrnes and Hayes [51] discovered that those with a heightened capsaicin burning threshold consumed spicy meals more often and assessed the burning sensation more favourably. Hayes et al. [177] found positive correlations between perceptual responses to piperine, zingerone, and capsaicin; Törnwall et al. [144] and Nolden and Hayes [100] also showed that high sensation seeking tendencies were associated with more positive evaluations and more frequent consumption of capsaicin-rich foods.

Research that elucidates how variations in sensation seeking affect the enjoyment received from habitual spicy food consumption provides personality-centered frameworks informed by psychophysiological and cultural viewpoints on desensitisation and sensory adaptation. Terasaki and Imada [178] conducted a study involving 105 university students, revealing that individuals with elevated ‘Sense Seeking Scale’ (SSS) scores exhibited a greater preference for spicy foods, meats, and alcoholic beverages. Notably, the Thrill/Adventure Seeking and Experience Seeking subscales demonstrated a significant positive correlation with the inclination towards spicy foods (refer to [167]). Törnwall et al. [144] reported that individuals with a pronounced sensory-seeking inclination exhibit a greater affinity for spicy foods and find the burn of capsaicin more enjoyable, whereas those with a lower pain threshold display diminished liking; nonetheless, regular exposure may enhance this impression ([51] corroborated this pattern of results).

Variations in sensation seeking account for a substantial aspect of the hedonistic allure of hot pepper use, whereas behavioural studies elucidate how usage frequency affects both tolerance and desire. Prescott and Swain-Campbell [167] demonstrated that those individuals with a pronounced propensity for sensation-seeking rated capsaicin-laden foods more positively and exhibited a greater likelihood of consuming them. Byrnes and Hayes [13] indicated that men exhibit more spice consumption and had a higher tolerance for capsaicin than women, potentially linked to biological sensitivity and correlations with masculinity. Bègue et al. [179] indicated a positive correlation between “macho” personality qualities and an affinity for chilli peppers, but Alley and Burroughs [180] found that men exhibited greater interest in spicy foods and were more inclined to experiment with foreign cuisines than women.

**Figure 5:**
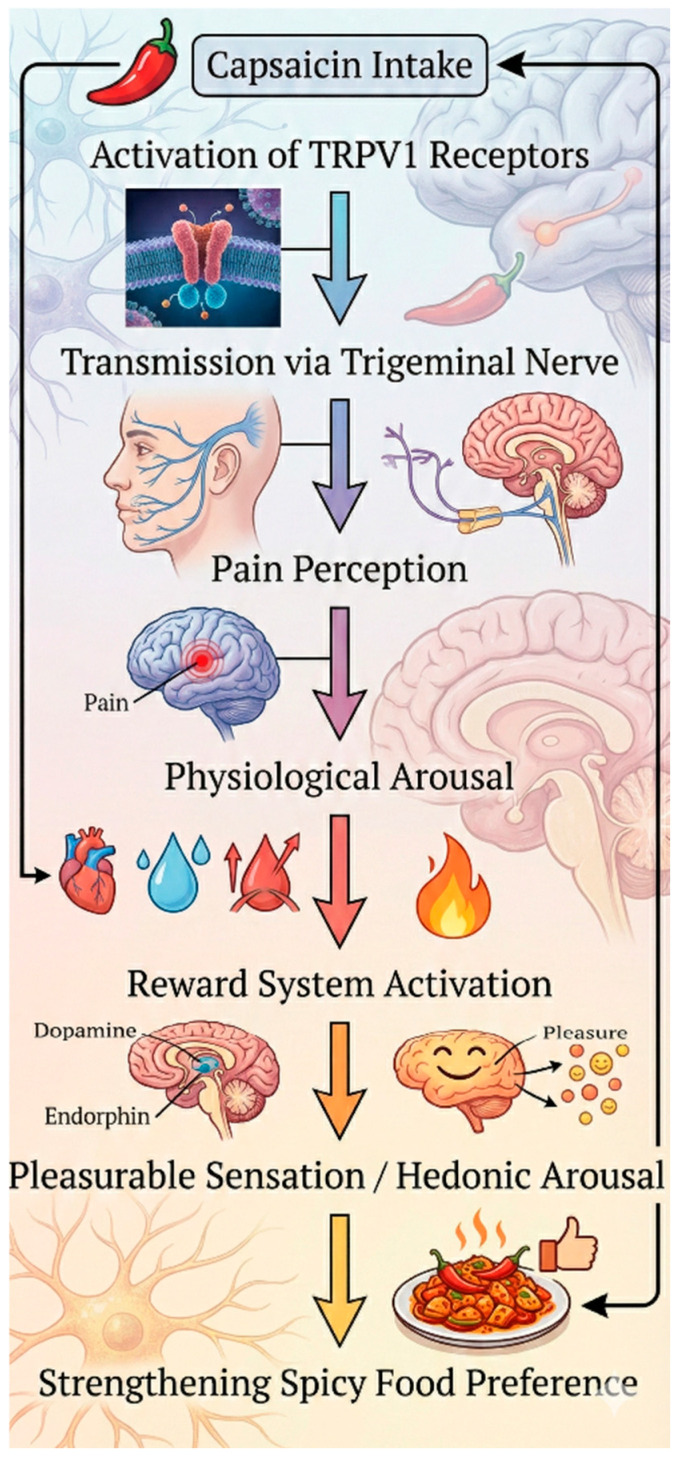
Sequential process of capsaicin-induced stimulation, physiological arousal, and dopaminergic reward activation reinforcing spicy food preference.

Conversely, not all individuals are attracted to pungency; Personality traits and emotional circumstances may also contribute to the aversion of spicy and unfamiliar meals. To explore this behavioural variability, Wang et al. [53] investigated the correlation between preference for spicy tastes and a propensity for risk-taking across three studies. In Study 1, persons with a preference for strong pungent stimuli were regarded as more risk-seeking, indicating that flavour choices may reflect fundamental psychological traits. In Study 2, those with a propensity for pungent/spicy cues showed elevated risk-taking scores on the Domain-Specific Risk-Taking Scale (DOSPERT-C), substantiating this behavioural connection with psychometric evidence. In Study 3, the consumption of spicy food in a laboratory setting correlated with riskier decisions on the Iowa Gambling Task, demonstrating the same effect under experimental conditions. Collectively, this research offers corroborative evidence that a preference for pungent or spicy stimuli correlates with risk-oriented personality traits. Wang et al. [53] posited that this association may be elucidated by the ‘benign masochism’ concept, wherein the physiological arousal induced by capsaicin ingestion stimulates the dopamine-mediated reward system and heightens risk-taking propensities (see Figure 5).

Furthering this area of research, a multi-national study including 8906 consumers reported that individuals exhibiting strong food neophobia consistently evaluated chilli-containing and other spicy foods and culturally diverse products more negatively [181]. This finding suggests that the intense trigeminal (chemesthetic) stimulation elicited by chilli peppers may trigger more negative affective responses among people with high food neophobia. In addition, high neuroticism scores have been shown to be significantly associated with a preference for spicy flavours [182]. Data from 224 university students revealed a favourable correlation between neuroticism and preferences for spicy dishes. The findings may be elucidated by the tendency of neurotic individuals to want more intense and stimulating experiences as a means of managing unpleasant emotions and stress [182]. The complex characteristics of spicy food desire can be examined through biological, psychological, and personality dimensions, correlating neurochemical activation with emotional and behavioural reactions (see Table 1).

Recent experimental research has examined the interplay between spicy flavours and emotional cognition and arousal, extending the discourse from avoidance tendencies to affective processing. In a three-stage experimental investigation reported by Chen et al. [183], individuals with a pronounced taste for spicy food exhibited heightened sensitivity to facial emotions of wrath and disgust. The link was influenced by personality-driven aggression tendencies and pathogen avoidance tendencies. Moreover, it was shown that the consumption of spicy food in experimental settings elevated participants’ state aggression levels, thereby enhancing their perception of facial displays of rage. The findings indicate that spicy flavours provoke physiological arousal and have complex effects linked to individual personality factors and emotional cognition processes [183].

Experimental findings associate spiciness with emotional arousal, while cross-cultural viewpoints emphasize its role as a symbolic representation of strength, courage, and social dominance. Heidari et al. [184] assert that the preferences for spicy cuisine correlates with specific personality characteristics. Specifically, the consumption of spicy peppers has been correlated with attributes such as strength, courage, and masculinity across various cultures; these inclinations are also associated with heightened sensation seeking, a predisposition to anger, and testosterone-related social dominance behaviours. Research suggests a potential correlation between men’s testosterone levels and capsaicin, the principal component of chilli peppers. A study identified a favourable association between individuals’ desired quantity of spicy sauce in a laboratory environment and their endogenous testosterone levels, assessed using saliva samples from men aged 18 to 44 years. This indicates that males with elevated testosterone levels are inclined to favour spicier foods; nevertheless, it does not establish that chilli peppers increase testosterone levels [179].

Apart from its symbolic and gendered connotations, the liking for spicy foods has been associated with openness, curiosity, and a propensity for sensory exploration. A comprehensive study by Robinson et al. [185] with 2034 participants highlighted a strong correlation between the consumption of spicy foods and the desire for variety amongst consumers. The research indicated that younger persons and Hispanic/Latino consumers, specifically, demonstrated greater interest in both spicy snacks and fresh chilli peppers. This finding indicates that preferences for spicy food may be attributed to consumers’ receptiveness to novel experiences and personality-driven motivations [185].

Historically and cross-culturally, the allure of pungency is seldom attributable to simple desire; instead, it embodies complex biological, cultural, and pharmacological interactions. Abdel-Salam [186] asserts that the demand for spicy pepper arises from a multifaceted interplay of biological, cultural, and pharmacological influences. Nabhan [187] proposed that capsaicin tolerance may confer functional benefits, such as diminishing infection risk in hot areas; thus, it aligns with cultural and genetic adaptations. Spence [29,39,102] investigated the cultural, psychological, and biological foundations of hot pepper consumption through a multisensory perception lens; he proposed that factors such as acquired preferences, social learning, risk propensity, sensory exploration, and endorphin release may affect consumption.

Studies have expanded on these personality-based findings by investigating how emotional characteristics, particularly anger and arousal, influence the experience and evaluation of spicy foods. Taken together, the evidence clearly suggests that adult food preferences are shaped, at least in part, by personality factors. Prior research indicates that sensation-seekers tend to ‘like it spicy’ (and possibly also sour and crunchy; e.g., [146,166,188]). Moreover, individuals with a significant inclination for thrill-seeking are inclined to pursue more intense stimuli, which correlates with a heightened interest in spicy foods [51,181]. Khan et al. [189] examined college students with diverse personality qualities (e.g., extraversion, agreeableness) and discovered a positive correlation between pleasant moods and preferences for spicy foods. They observed that distinct personality qualities exhibited strong relationships with particular preferences for spicy foods [189].

Ji et al. [190] examined the cross-cultural association between rage and spicy taste in regard to metaphor and corporeal theory. The findings suggest that individuals who relish spicy foods are more prone to exhibit anger in entirely anonymous contexts, and that trait anger assessments are positively associated with preferences for spicy food. These findings correspond with the current research detailing the association between sensory desire and spicy foods [115,189]. Although anger-related theories provide one viewpoint, additional emotional and motivational reasons may also influence the attraction to spicy meals. Unpleasant emotions and arousal during consumption may provoke aggressive attitudes or thoughts in some individuals [191]. The association between anger and spice has received limited to moderate empirical validation [190]. Research suggests that the consumption of spicy meals cannot be ascribed to a single motivational reason (e.g., violence), while positive emotions amplify the preference for spiciness [189]. The interaction of sensory seeking, mood polarity, and personality is a significant factor [189,190].

Sensitivity to capsaicin (threshold and/or suprathreshold intensity) was found to be reduced in frequent consumers of spicy foods, while sour taste and intranasal trigeminal sensitivity remained unchanged. This suggests that habituation in daily life and inducible desensitization observed in the laboratory converge on the same trajectory and suggests that the exposure cycle may operate more rapidly in individuals with high spicy preference and high sensation seeking [26,145,192].

Collectively, these experimental and observational results, alongside previous personality studies, suggest that the liking for spiciness is best explained by a dual-process framework;

- a rapid, state-dependent layer, encompassing acute sensitisation and desensitisation dynamics in response to immediate stimulation [174,175]; and
- a slower, personality-modulated layer, in which repeated exposure produces more chronic desensitisation and a progressive cycle of acceptance and preference influenced by sensation seeking and mood [26,192].

The inclination towards spicy foods signifies an adaptive interaction among biology, personality, and culture. Physiological desensitisation and psychological habituation illustrate how repeated sensory experiences can alter hedonic assessment. This dynamic renders the perception and consumption of spicy foods a distinctive framework for examining the intersection of psychology, neuroscience, emotion, and behaviour in consumption selection).

## 5. Conclusions

The consumption of chile is an acquired behaviour influenced by personality traits such as sensation seeking and reward sensitivity, as well as by the hedonic properties of pungent stimuli [26,51,63,100,144,177,189,193]. This intersection has yet to be examined in a systematic and cross-cultural manner within the literature. The research that has been reviewed here suggests that the desire for spicy foods cannot be exclusively ascribed to biological sensitivity. In fact, the research that has been published to date illustrates a dynamic interaction between cultural learning, social reinforcement, and characteristics of an individual’s personality. Recent studies demonstrate that the burning sensation that can be induced by capsaicin and analogous trigeminal stimuli elicits varying hedonic responses amongst people. Genetic factors, psychological characteristics, and cultural influences may account for this difference. Nonetheless, these connections have predominantly been examined in restricted groups, there remains a clear need for comprehensive research across diverse cultural contexts.

Certain individuals like spicy cuisine, while others do not. These differences in hedonic response reflect a combination of biological sensitivity, personality-related factors, and prior cultural experiences. This helps to explain why spicy foods may become integrated into the everyday diets of some people, while consumption remains comparatively limited among others. Future research ought to investigate the biological, psychological, and social correlates of spicy-food consumption, with particular emphasis on personality dimensions beyond sensation seeking and reward sensitivity. Examining additional traits-such as impulsivity or broader domains within the Big Five-would help clarify whether spicy-food preference reflects a general personality profile or a more specific behavioural disposition linked to identity, culture, and sensory experience.

## Figures and Tables

**Table 1:** Conceptual mapping of domains, constructs, and underlying mechanisms associated with spicy food preference (selected evidence).

Domain	Construct	Conceptual Definition	Underlying Mechanism	Representative Evidence
Biological (Neuro)	TRPV1 activation	Capsaicin stimulates heat/pain receptors, producing a burning sensation	TRPV1 channel; trigeminal pathway-central processing	[58,112,113]
Biological (Neuro)	Trigeminal-reward linkage	Physiological arousal from pungency links to reward responses	Trigeminal afferents- dopaminergic system	[27,36,119]
Biological (Adaptation)	Sensitization/desensitization	Repeated exposure alters burn perception (decrease/recovery)	Peripheral desensitization	[25,76,149,151]
Multisensory Integration	Crossmodal modulation	Pungency changes taste/aroma perception and release	Taste–odor–trigeminal integration	[42,43,70,102]
Physiological	Energy/metabolic effects	Spicy foods influence thermogenesis and energy balance	Autonomic/thermogenic responses	[56,115]
Psychological (Risk)	Risk-taking tendency	Disposition to seek high-arousal, uncertain situations	Reward sensitivity/sensation seeking	[17,53,147]
Personality	Neuroticism and neophobia	Emotional instability and avoidance of novelty/discomfort	Amygdala-driven stress/threat response	[11,12,34]
Individual Differences	Habitual spicy users	Regular consumers show distinct sensory/psychosocial profiles	Desensitization; learned reinforcement	[26,40,71,115]
Behavioural Outcomes	Preference/acceptance	Liking and choice of pungent foods	Hedonic arousal; reinforcement learning	[13,50,143,145]
Developmental	Exposure-based learning	Preference acquired via prenatal/early and repeated exposures	Flavor transfer; mere exposure effects	[62,77,124,154,155]
Genetics	Bitter/TAS2R and heritability	Genetic variation shapes taste and pungency-related responses	TAS2R38 variants; twin/heritability evidence	[4,8,10,18,19,144]
Cultural-Historical	Domestication and diffusion	Origins, domestication, and global spread of chili peppers	Genetic/archaeobotanical lines of evidence	[84,85,87,88,90,94,118]
Cultural-Societal	Culinary globalization	Why some societies embrace “the burn”	Migration, acculturation, culinary identity	[60,61,95,96,97,102,116]
Media and Marketing	Spicy challenges and influence	Media-driven diffusion of extreme spiciness and risk	Influencer/advertising effects; challenge culture	[78,131,132,133,134,135,137]
Measurement	Chemesthesis/taste tools	Measuring pungency/capsaicinoids and temporal burn	HPLC; electrochemical sensors; TI/TCATA	[49,105,107,110,141]
Pharmacology and Pain	Analgesia and neuropathic pain	Capsaicin modulates pain pathways and analgesia	TRPV1 modulation/ablation	[47,119,153]
Social-Affect	Spicy-emotion links	Pungency relates to affect, cognition, and metaphor	Emotional regulation; metaphorical mapping	[14,52,114,148]
Sensory Physiology	Thresholds and sensitivity	Individual/regional differences in oral irritation	Regional sensitivity; thickness/heat thresholds	[23,45,150]

## Data Availability

No new data were created or analyzed in this study. Data sharing is not applicable to this article.
